# A 3D-Printed Portable UV and Visible Photoreactor for Water Purification and Disinfection Experiments

**DOI:** 10.3390/nano14060525

**Published:** 2024-03-15

**Authors:** Nelson Castro, Joana M. Queirós, Dinis C. Alves, Margarida M. Macedo Fernandes, Senetxu Lanceros-Méndez, Pedro M. Martins

**Affiliations:** 1 Physics Centre of Minho and Porto Universities (CF-UM-UP) and LaPMET—Laboratory of Physics for Materials and Emergent Technologies, University of Minho, 4710-057 Braga, Portugal; nelson.castro@inl.int (N.C.); id10031@alunos.uminho.pt (J.M.Q.); dinisalves@fisica.uminho.pt (D.C.A.); margaridafernandes@cmems.uminho.pt (M.M.M.F.); 2 International Iberian Nanotechnology Laboratory (INL), 4715-330 Braga, Portugal; 3 Centre of Molecular and Environmental Biology, University of Minho, 4710-057 Braga, Portugal; 4 IB-S—Institute for Research and Innovation on Bio-Sustainability, University of Minho, 4710-057 Braga, Portugal; 5 Center for Microelectromechanical Systems (CMEMS) and LABBELS—Associate Laboratory, University of Minho, 4800-058 Guimarães, Portugal; 6 BCMaterials, Basque Center for Materials, Applications and Nanostructures, UPV/EHU Science Park, 48940 Leioa, Spain; 7 IKERBASQUE, Basque Foundation for Science, 48009 Bilbao, Spain

**Keywords:** portable photoreactor, photocatalysis, ciprofloxacin, water purification, 3D printing

## Abstract

Water scarcity and contamination are urgent issues to be addressed. In this context, different materials, techniques, and devices are being developed to mitigate contemporary and forthcoming water constraints. Photocatalysis-based approaches are suitable strategies to address water contamination by degrading contaminants and eliminating microbes. Photoreactors are usually designed to perform photocatalysis in a scalable and standardised way. Few or none have been developed to combine these characteristics with portability, flexibility, and cost effectiveness. This study reports on designing and producing a portable (490 g), low-cost, and multifunctional photoreactor that includes adjustable radiation intensity and two types of wavelengths (UV-A and visible), including combined agitation in a compact mechanism produced through 3D printing technology. The mechanical, electrical, and optical subsystems were designed and assembled into a robust device. It is shown that it is possible to apply radiations that can reach 65 mW/cm^2^ and 110 mW/cm^2^ using the installed visible and UV LEDs and apply mechanical agitation up to 200 rpm, all under a ventilated system. Regarding functionality, the photoreactor proof of concept indicated the ability to degrade ~80% and 30% ciprofloxacin under UV and visible irradiation of TiO_2_ and Ag/TiO_2_ nanoparticles. The device also showed the ability to eliminate *E. coli* bacteria, recurring to radiation set-ups and nanoparticles. Therefore, the originally designed and constructed photoreactor concept was characterised and functionally validated as an exciting and flexible device for lab-scaled or outdoor experiments, assuring standardised and comparable results.

## 1. Introduction

Water contamination is a planetary-scale problem driven by several factors, such as industrial activities, agricultural practices, population density, and societal habits, that demands urgent action to reduce its harmful effects on aquatic organisms and human health. The United Nations members have identified clean water as the sixth goal of the Sustainable Development Goals. The World Health Organisation estimates that approximately 800 thousand people die yearly from illnesses related to contaminated water consumption [1,2]. 

In this critical context, there are increasing efforts to develop new methods, materials, and devices that can efficiently decontaminate water [3].

As a result, research on advanced oxidation processes (AOPs) for water purification has gained significant attention as an alternative and complement to conventional water treatment methods. These innovative techniques facilitate the breakdown of various organic contaminants by generating highly reactive species [4,5,6,7].

AOPs have paid much attention to the photocatalytic processes to degrade organic contaminants in water matrixes [8,9,10]. The photocatalyst more commonly used is titanium dioxide (TiO_2_), owing to its advantageous properties, such as low toxicity, abundance, cost effectiveness, and chemical stability [11]. To efficiently perform photocatalytic degradation processes in a well-controlled, scalable, and reproducible way, it is necessary to design and develop photoreactors. These devices should allow the photons from a radiation source to be in contact with the photocatalysts (e.g., titanium dioxide (TiO_2_)) and the contaminant in the water matrix under continuous stirring for homogenisation, allowing for initiation and maintaining the photocatalytic degradation process [12]. 

Typically, the photocatalytic reactors presented in the literature can be divided into two main types: a reactor with the photocatalyst (e.g., TiO_2_ nanoparticles) in suspension and a configuration with immobilised photocatalysts. The main difference between those is the catalyst placement: in suspension reactors, the catalyst is suspended in a reactant solution; in the other, a catalyst is immobilised in the reactor walls or on a substrate (e.g., zeolites, polymer), where the reactant solution flows through [13]. The former presents higher efficiencies, as the nanoparticle’s entire surface area is available for radiation harvesting and interaction with the organic contaminants in suspension, and it is usually the first stage that allows the evaluation of newly produced photocatalysts. On the other hand, it demands post-processing for nanoparticle removal to prevent secondary pollution, which makes decontamination more expensive [14]. The latter type of reactor immobilises nanoparticles that show significant photocatalytic degradation efficiencies into a substrate. This enables their reuse and the reduction in post-processing steps and costs. It also prevents secondary pollution with the trade-off of lower photocatalytic efficiency due to decreasing the nanoparticle’s active contact area [14].

Photoreactors with very different configurations have been produced; for instance, an immobilised solution involving a tubular photoreactor based on cellulose acetate monolithic/TiO_2_ thin films has been irradiated with simulated or natural solar light, removing approximately 100% of Cr in the solution [15]. Another immobilised solution developed a fixed-bed reactor utilising 12% wt. TiO_2_-coated alumina monoliths incorporated in the space between a centrally deployed UV lamp and the reactor wall to degrade 1,8-Diazabicyclo[5,4,0]undec-7-ene, obtaining 100% conversion [16].

Regarding suspension reactors, Khawla Azalok et al. photodegraded 98% of metronidazole in 60 min, using TiO_2_ as a catalyst under UV irradiation [17]. Further, García-Muñoz et al. used a batch suspension reactor with TiO_2_ as a catalyst to degrade polystyrene nanobeads (PS); this work showed the removal of approximately 100% of 20 ppm PS 6 h after the beginning of the experiment [18].

Additionally, water disinfection applications have also been reported, with the inactivation of 92.6% of Escherichia coli [19] after 120 min of visible irradiation over polystyrene spheres with nitrogen-doped TiO_2_ particles and 100% using 0.6% wt. Ag/TiO_2_ nanoparticles [20]. 

To evaluate photoreactors and among the many possible contaminants, one category of pollutants deserves particular attention: the contaminants of emergent concern (CECs). These substances can be pharmaceuticals, personal care products, or pesticides that are not regularly monitored in the environment. However, they can potentially pose negative ecological and health consequences for humans [21,22], and conventional wastewater treatment techniques cannot effectively remove these CECs [23].

Further, the aquatic environment provides a thriving environment for microorganisms such as enteric bacteria, viruses, protozoa, and parasitic worms [24,25]. Public health authorities often prioritise pollutants that cause immediate illness and the spread of diseases, particularly regarding non-potable wastewater recycling [26]. Nevertheless, it has been demonstrated that traditional disinfection methods, such as chlorine disinfection, are inadequate and have led to resistant bacterial strains, posing a significant health risk in water-related applications [27].

Based on the mentioned state of the art, regarding both photoreactors for contaminant degradation and disinfection, there is a vast diversity of devices whose control of parameters such as radiation intensity, type of radiation, container (volume), and agitation are difficult to vary, control and, therefore, make them reproducible, aiming to create comparable scientific data. In this context of photocatalytic assays, there is a demand for new and standardised approaches that allow for straightforward comparisons between results.

In this work, the focus was on the development of a new concept of a portable photoreactor for lab-scale photocatalytic assays under UV or visible radiation with an articulated stirrer connected to a motor. The prototype parts were produced by FDM (Fused Deposition Modelling) 3D printing, and the circuits were designed for PCB (Printed Circuit Board) construction. The constructed photoreactor functionalities were validated regarding its photocatalytic and disinfection properties using well-known photocatalysts (TiO_2_ and Ag/TiO_2_) to evaluate a model contaminant’s (ciprofloxacin—CIP) photocatalytic degradation and efficiency against *E. coli*, both under UV and visible LED irradiation installed in the photoreactor. The suitability of this reactor for the assessed functionalities was proved, and the ability to standardise and tune experiments was equally reassured. 

## 2. Materials and Methods

### 2.1. Photoreactor Design and Fabrication

- Mechanical subsystem

The system was designed as a small portable actuation device of 144 × 83 × 149.5 mm (L × W × H), which enables LED irradiation to 50 mm diameter beakers used with the contaminated solutions to be tested with UVA or visible radiation. 

**Figure 1:**
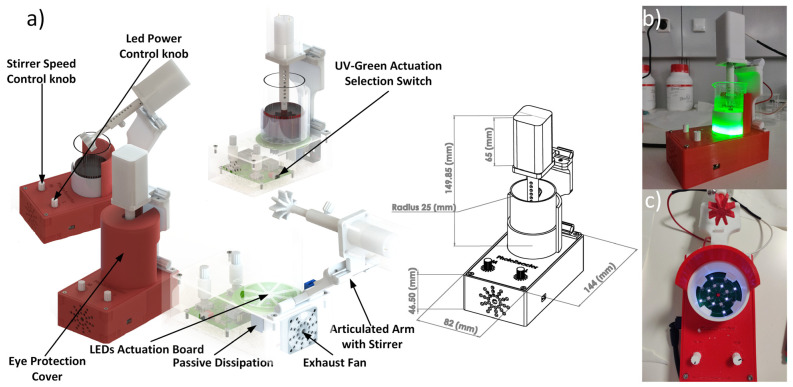
A schematic representation of (**a**) the external and internal perspectives of the mechanical appearance of the 3D-printed photoreactor fully assembled, and all device measurements are in mm. The real pictures represent the (**b**) lateral and (**c**) superior perspectives of the photoreactor displaying VIS (green) and UV (purple) radiation turned on.

For this purpose, to reduce power consumption and improve light transmission efficiency, light actuation was applied from the bottom. However, this architecture hinders a magnetic stirrer at the bottom of the beaker, requiring a mechanical stirrer placed at the top. In Figure 1, the schematic representation of the device is shown where the stirrer attached to the motor was designed to be articulated, allowing for its insertion or removal from the reaction beaker (100 mL) that contains the contaminated solution and the photocatalysis (e.g., titanium dioxide nanoparticles). Furthermore, the stirrer is easily withdrawn and washed at the end of each use. 

A removable printed cover protects the user’s eye from the UVA and visible irradiation. A side switch provides the control with three positions: no light actuation, visible light, or UVA light. The user interface is a simple control of the stirrer rpm (rotations per minute) and light intensity, which relies on the PWM (Pulse Width Modulation) signal manipulation, which actuates the inner electronics. The mechanical parts were designed using SolidWorks 2017^®^ as CAD software, which enabled the possibility of 3D printing them using a Prusa^®^ iMK3 printer with PLA (polylactic acid filament—a Prusament reference from Prusa, Prague, Czech Republic). Further, a conventional 200 rpm, small DC motor (FIT0492 from BDFROBOT) was installed and screwed to the printed structure to apply mechanical stirring to the features of the device. The final assembled device weighs 490 g, which perfectly matched the intentions of developing a lightweight device that allows for easy transportation (e.g., field experiments) and high practicability and flexibility (e.g., lab scale assays) for the photocatalytic and disinfection assessment set-ups.

- Electrical subsystem

**Figure 2:**
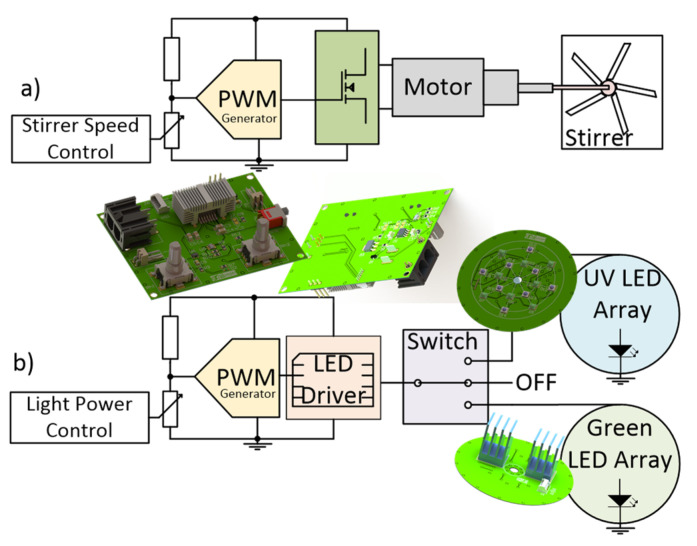
Simplified circuit diagrams for the control of (**a**) the motor stirrer by a PWM signal being applied to a field-effect transistor followed by the motor, and (**b**) an LED driver receives the PWM signal and applies a current stream to the LED array.

The electrical design of the photoreactor is summarised in Figure 2 with the simplified circuit diagrams. Two potentiometers (RK1191124001 from Alps Alpine, Tokyo, Japan) are instrumented with a reference resistor (standard surface mount 0805 sizes) and the respective DC voltage variation, received by an LTC6992 from Analog Devices (Wilmington, MA, USA) and transduced into a PWM signal accordingly. The transduction happens the same way for both system components; however, the following circuits are different: in Figure 2a, a MOSFET receives the signal in the gate pin, which enables the power drawing from the source by the motor (maximum 200 rpm) and thus actuating the stirrer mechanically coupled; in Figure 2b, a dedicated LED driver integrated circuit with reference 172946001 from Wurth Electronics (Yokohama, Japan), receives the PWM signal and accordingly applies a controlled current throughout the LED array PCB. 

**Figure 3:**
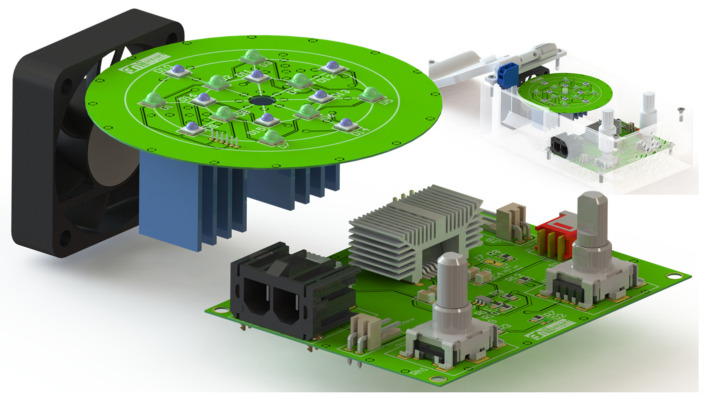
Schematic representation of the photoreactor PCB’s disposition according to passive and active heatsink systems.

However, transforming electrical power into light generates heat in the system, requiring both passive and active heatsink components. The heat dissipation is critical to maintaining the stability of the electrical part, but also because the system’s main structure is 3D printed in PLA, which could melt in the vicinities of the heatsinks and LEDs. Thus, as shown in Figure 3, the heatsink for the LED driver was based on encapsulation DA-T263-401E-TR from Ohmite (Warrenville, IL, USA), and for the LED array, on a PCB holding ATS-54150W-C1-R0 from Advanced Thermal Solutions (Norwood, MA, USA). 

Regarding active heat dissipation, a 12 V 40 mm conventional fan (CFM-4010B-180-296-22 from CUI Devices, Lake Oswego, OR, USA) and frontal and back air entrances were installed for air circulation around the passive heatsinks. Furthermore, the system uses only one of the two arrays of LEDs to keep power dissipation at lower levels.

- Irradiation subsystems

The eight UV and eight visible LEDs were installed, separated by approximately 15 mm between them. The LEDs’ voltage was tuned by dividing 30 V by eight LEDs, yielding 3.75 V for each. The wavelength of the LEDs matches the typical semiconductor catalyst’s absorbance peaks in the UVA region, namely 365 nm (SST-10-UV from Luminus, Sunnyvale, CA, USA). The effective radiation source range depends on the photocatalyst; for TiO_2_, the light absorption is in the ultraviolet range, up to 400 nm [28]. The ultraviolet radiation spectrum is divided into three zones depending on the wavelength: long wave (UV-A) from 315 to 400 nm, middle wave (UV-B) from 280 to 315 nm, and short wave (UV-C) from 200 to 280 nm [29]. Traditional tube technologies for UV-B and UV-C radiation have drawbacks, such as the need for expensive high voltages, fragility, and the use of mercury. This radiation is also associated with skin and eye problems [30,31,32]. In this context, to make the process cheap, practical, and safe in this work, LED-based UV-A technologies emitting a radiation of 365 nm are adequate and readily commercially available [33]. Concerning visible range radiation, the LED peak of 535 nm (XPEBGR-L1-0000-00G03 from Cree XLamp XP-E2) was installed to allow for the assessment of the ability of nanocatalysts (e.g., Ag/TiO_2_) to absorb the visible range radiation to perform photocatalysis. The device’s structural design provides up to 450 mA flow in the LED arrays, with a controlled PWM of 800 kHz up to 80% (hardware limited). 

**Figure 4:**
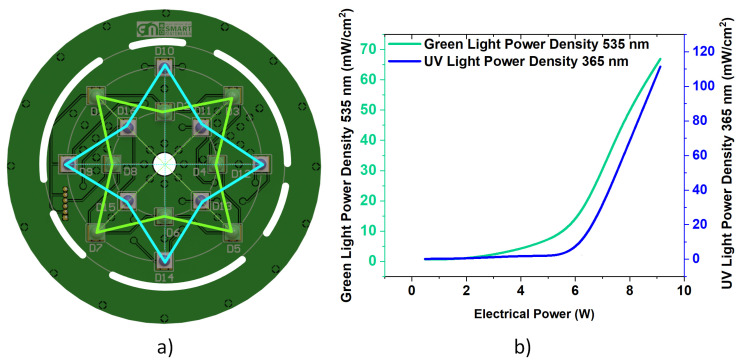
Light power is produced from both LED arrays according to the electrical power applied. (**a**) Mechanical disposition of both types of LED with UV and Visible light intertwined for a similar light actuation disposition and (**b**) Ratio of light intensity for each Wavelength type according to applied power.

The photocatalytic degradation of ciprofloxacin and the disinfection processes are driven by irradiation. Therefore, the LED disposition was carefully studied to allow for UV and visible irradiation through the device and simultaneously allow for uniform and efficient photocatalytic and disinfection processes. The four-pointed star arrangement for UV and visible LEDs (Figure 4a) allows for a uniform irradiation of the bottom of the beaker, assuring that solutions containing the nanocatalysts and the contaminant are entirely and equally irradiated. It is equally important to quantify photo actuation from the device. The light actuation of both LED arrays was measured at a constant 5 mm distance from the bottom to the beaker using an S425C surface absorber from Thorlabs, Figure 4b.

The electrical power drawn by the LEDs is controlled by the PWM signal in the LED driver set by the potentiometer position, and a similar approach controls the motor rpm. Regarding the light actuation results, it is realised that below 5 W of electrical power applied to a single array of LEDs yields no significant light irradiation (less than 10 mW/cm^2^) for both collections. Concerning UV radiation (365 nm), 6 W produces ≈7.5 mW/cm^2^ power density, increasing to ≈110 mW/cm^2^ with 9 W of electrical power. Additionally, the visible light (535 nm) produced by applying 6 W of electrical power is ≈14 mW/cm^2^ and reaches 66 mW/cm^2^ for 9 W. It is relevant to stress that the electrical power of the LEDs was in part limited by the electric-to-light transformation, presenting plenty of heat loss. In a miniaturised and portable device, electrical power/radiation intensity was sacrificed to maintain heat dissipation at secure levels. 

### 2.2. Functional Performance Evaluation of the Photoreactor

**Figure 5:**
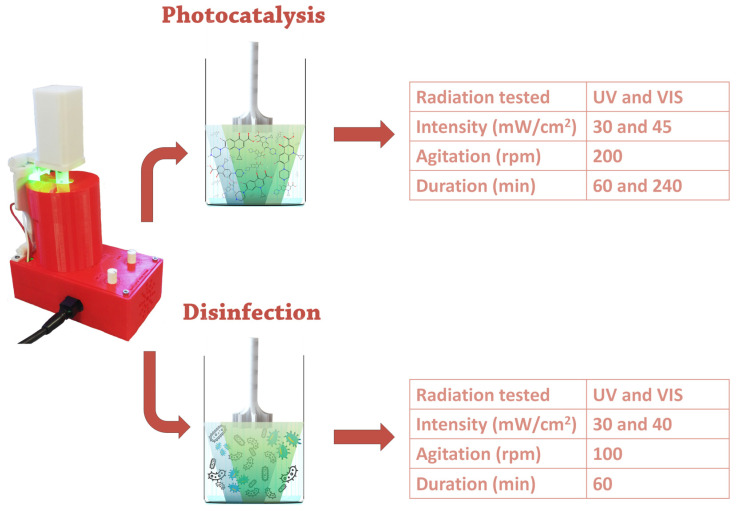
A schematic representation of the functional tests to evaluate the photocatalytic degradation of ciprofloxacin and the disinfection of *E. coli* with the designed and assembled photoreactor. Tables list the experimental conditions employed in each assessment.

After the complete assemblage of all components, functional tests to evaluate the photocatalytic and disinfection features of the device were performed. Figure 5 emphasises the versatility of the developed photoreactor, which allows for tuning the type of radiation (UV-A or visible range), its intensity, and agitation rate in a standardised, reproducible, comparable manner usually lacking in photocatalytic assessments.

In this way, many applications, such as the photocatalytic degradation of contaminants (e.g., ciprofloxacin) or microbial disinfection (e.g., *E. coli*), can be investigated with the proposed multifunctional system. Photocatalytic nanoparticles (TiO_2_ and Ag/TiO_2_) were employed during both functional tests. It is essential to highlight that the nanocatalysts used in these photocatalytic tests were already synthesised, characterised, and applied to remove contaminants and bacteria [9,34,35]. The role of these materials in this work is to validate the developed photoreactor concept. The portability allows for experiments in the field or laboratory, and the flexibility enables tuning parameters such as radiation type, intensity, agitation, and exposure time, as demonstrated in Figure 5. 

#### 2.2.1. Photocatalytic Assays

Commercial titanium dioxide nanoparticles (TiO_2_), with a surface area ranging from 35 to 65 m^2^/g, were acquired from Evonik Industries AG. The Ag/TiO_2_ nanocomposites were produced by the deposition–precipitation method, as reported in [35]. Ciprofloxacin (Mw = 331.34 g/mol) with a maximum absorption of 277 nm was purchased from Sigma-Aldrich (St. Louis, MO, USA). 

The photocatalytic activity of TiO_2_ and Ag/TiO_2_ was evaluated by monitoring the degradation of ciprofloxacin under ultraviolet (UV) and visible (VIS) LED radiation. A standard solution of CIP (5 mg L^−1^, pH = 2.9) was prepared. Then, to 50 mL of this solution, 50 mg of the catalyst was added and kept under agitation in the dark to reach adsorption/desorption equilibrium. Subsequently, the solution was exposed to UV radiation to test the photocatalytic activity of TiO_2_ nanoparticles. Similarly, the solution was exposed to VIS radiation to test the photocatalytic activity of Ag/TiO_2_ nanoparticles. The photocatalytic degradation efficiencies of CIP were analysed by measuring the maximum absorption peak (277 nm) over time using an Infinite M Plex spectrophotometer (TECAN). The degradation fits the Langmuir–Hinshelwood model, expressed by Equation (1):

(1) $$\frac{C}{C_{0}}=e^{-kt}$$

where $C_{0}$ and C are the initial concentration of the pollutant and the concentration at time (t), respectively, and k is the first-order rate constant of the reaction (min^−1^) [36]. 

#### 2.2.2. Antimicrobial Assays

Gram-negative Escherichia coli ATCC^®^ 8739™ were purchased from the American Type Culture Collection (LGC Standards S.L.U, Barcelona, Spain). Bacterial pre-inoculum was prepared using a single colony from the corresponding stock bacterial culture, resuspending it in nutrient broth (NB), and then incubating overnight at 37 °C and 110 rpm. After 20 h, the bacteria were harvested by centrifugation at 4500 rpm for 5 min and resuspended in NaCl 0.9% (*w*/*v*) twice. The *E. coli* culture optical density (OD) was adjusted to OD = 0.26, measured at 600 nm, giving rise to a working inoculum of approximately 1 × 10^7^ colony-forming units (CFU) per mL.

The bactericidal activity was assessed according to the standard shake flask method (ASTM-E2149-01) with some modifications as an adaptation to the photoreactor set-up. This method provides quantitative data for measuring the reduction rate in the number of *E. coli* colonies formed, converted to the average colony-forming units per millilitre of buffer solution in the flask (CFU/mL). E. coli eradication was assessed using the developed photoreactor with two different radiation sources. 

The UV radiation was tested in the presence of the bacterial inoculum at 37 °C for one hour (the control was the same bacterial inoculum without applying UVs) without any particles to evaluate the effect of UV radiation on the bacteria viability. The VIS (green) radiation was also tested by mixing the solution with 0.5 mg/mL of Ag/TiO_2_ nanoparticles, previously synthesised and tested in other studies [34,35], to assess the synergy between the VIS radiation and the particles for efficient bacterial killing. The final volume of the solutions was 40 mL to fit the photoreactor. After one hour of incubation, the solutions subjected to the UV and green irradiation and respective controls were serially diluted (1:10) in sterile buffer solution, plated on a plate count NB agar, and further incubated at 37 °C for 24 h to determine the number of surviving bacteria. Antimicrobial activity is the percentage of bacteria log reduction calculated as the ratio between the number of surviving bacteria after and before the treatment (Equation (2)):

(2) $$Bacteria\;reduction\;\left(\%\right)=\frac{\left(A-B\right)}{A}\times 100$$

where *A* and *B* are the average number of bacteria before and after contact with the samples. The results were further expressed as a log_10_ reduction by calculating the log_10_ of bacteria reduction. All antibacterial data represent mean values ± SD (n = 3).

## 3. Results and Discussion

### 3.1. Photocatalytic Assays

**Figure 6:**
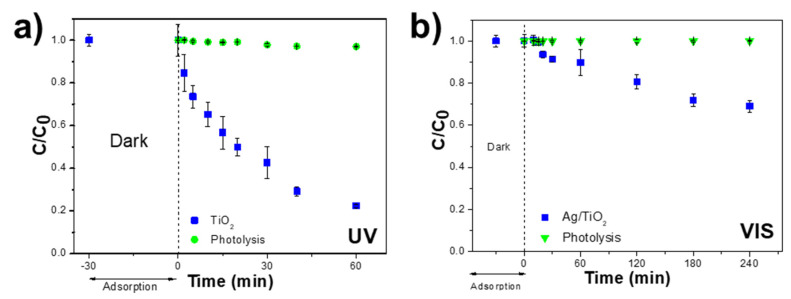
Photocatalytic degradation of CIP (5 mg L^−1^; pH ≈ 3; V_CIP_ = 50 mL) in aqueous solution under (**a**) UV radiation with TiO_2_-P25 (m_TiO2_ = 50 mg) particles for 60 min and (**b**) visible radiation with Ag/TiO_2_-P25 (m_Ag/TiO2-P25_ = 50 mg) nanoparticles for 240 min.

CIP degradation tests were performed under UV and VIS LED irradiation for 60 and 240 min to validate the developed photoreactor and its practicability in photocatalytic assays (Figure 6). 

The analysis of the photocatalytic experiments indicates negligible CIP adsorption by nanoparticles (~0%), suggesting a reliance on LED irradiation for pollutant removal. The contaminated solution was irradiated with UV and VIS LEDs in the photoreactor to estimate CIP photolysis. The results, shown in Figure 6a,b, show minimal degradation rates of approximately 3% and 0% of CIP under UV and VIS light, respectively. 

Afterwards, using TiO_2_ nanoparticles in contact with CIP solution under UV irradiation yielded a degradation efficiency of 78% and an apparent degradation rate constant of 0.024 min^−1^. Regarding the photocatalytic results obtained with Ag/TiO_2_ nanoparticles under VIS visible radiation, 31% of CIP in the solution was degraded with an apparent reaction rate of 0.002 min^−1^ obtained by applying Equation (1). These results indicate that irradiance energy plays an essential role in pollutant degradation. Therefore, as UV radiation is more energetic than visible light, it makes the valence electrons more easily excited so they to interact with the molecular species in the water, generating higher concentrations of reactive oxidative species (ROS). Therefore, this difference in degradation efficiency between UV and visible light tests can be attributed to the higher concentration of ROS generated by the former [37]. CIP was used as a model contaminant since its photocatalytic degradation process is already well known, with several works focused on the photocatalytic condition’s optimisation, photocatalytic degradation mechanism, and the toxicity of the generated by-products, among many others [9,38,39]. 

Despite the interesting photocatalytic results, the focus of this work was the validation of the design and the functional concept of the photoreactor, indicating that the bottom irradiation is efficient (intensity and spatial distribution), both from the standpoint of the photocatalytic degradation process, which was efficient, and in terms of the photoreactor stability, by avoiding excessive heating despite the prolonged irradiation (i.e., 1 and 4 h), and finally validating the agitation using an articulated mechanical stirrer. Additionally, it was possible to certify that the mechanical agitation provided is perfectly adjustable and in a range that allows for maintaining adequate nanoparticle dispersion in the CIP solution. 

Table 1 lists different studies focused on applying photocatalytic materials based on TiO_2_ with varying radiation sources for the degradation of antibiotics in an aqueous solution. Due to the remarkably different experimental conditions, such as pH, contaminant concentration, and radiation intensity/wavelength, direct comparisons cannot be made. This is one of the significant drawbacks of the photocatalytic experiments performed in the scientific community; a vast and infinite combination of experimental conditions hinders any direct comparison of the obtained photocatalytic degradation results. Nonetheless, the information is helpful for contextualisation purposes. 

The antibiotic degradation rates (*k*) related to the different materials and degradation conditions in Table 1 range between 0.02 and 0.001 min^−1^. Through an analysis of Table 1, this work presents a superior performance with similar experimental conditions. It is crucial to underline that most of the present works involve extensive, mono radiation, only UV or visible, non-portable photoreactors with higher irradiation intensities, yielding higher photocatalytic activities. The present photoreactor shows efficiencies interestingly similar to other systems in this context. But this device offers many features that others lack, such as UV and VIS radiation, adjustable agitation, portability, and standardised efficiencies that allow for direct comparisons if assessments are performed using the same conditions. Also, the idea of this photoreactor is not to compete with commercial devices in the market but to be a cost-effective, 3D-printable, and portable device that allows lab-scale (in lab or field) assessments to be performed before upscaled experiments. 

### 3.2. Antimicrobial Assays

**Figure 7:**
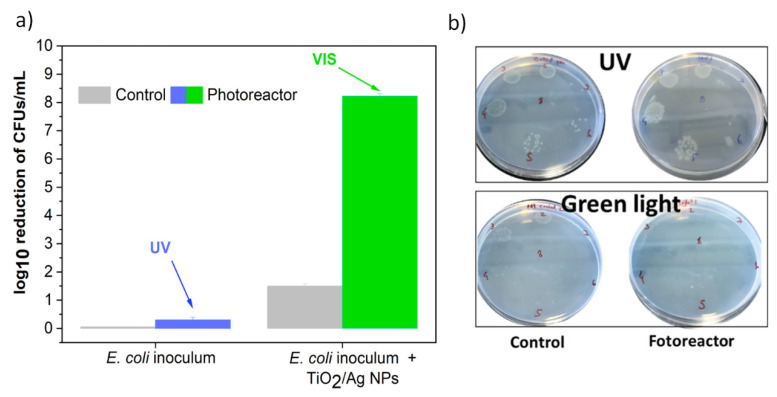
(**a**) The antimicrobial effect of the photocatalytic nanoparticles evaluated in the photoreactor applied to an *E. coli* inoculum using UV and green light, measured in a log_10_ reduction in CFUs; (**b**) Petri dishes displaying colonies that grew in nutrient broth agar.

The photoreactor was also tested on an *E. coli* bacterium, a microorganism commonly found in wastewater treatment facilities and usually a concern due to the risk of discharge into drinking water [43]. Thus, an alternative method to eliminate or inactivate these faecal bacteria through photocatalytic processes was tested in the developed reactor. Two different radiation sources were used: UV radiation directly on a bacterial inoculum to test the ability of UV irradiation to eliminate bacteria without the presence of nanoparticles, and VIS radiation on bacteria inoculated with 0.5 mg/mL of Ag/TiO_2_ to induce bactericidal effects through the activation of the ROS in these NPs due to the presence of Ag. The UV radiation has a negligible impact on reducing *E. coli* colonies (0.5 log_10_ reduction). At the same time, the NPs alone, without light, induced a 1.5 log_10_ reduction (Figure 7a) and represented colonies forming units (CFU) on Petri dishes in sequential dilutions (Figure 7b). The VIS green light from the photoreactor enhanced the bactericidal effect, yielding an impressive 8 log_10_ reduction, thus eliminating this bacterium. The VIS radiation applies wavelengths near the plasmon resonance of silver, which promotes the formation of ROS and changes the overall plasmon resonance of the Ag/TiO_2_ NPs, thus making it highly effective in killing bacteria [35]. During disinfection assays, the photoreactor showed irradiation stability and a suitable refrigeration system that prevented the heating of the contaminated water in the beaker. If the refrigeration was inefficient, temperature variations could have occurred and interfered with bacteria viability—cell death could be a consequence of the temperature and not the nanomaterial or irradiation. 

This approach may represent an essential breakthrough in the treatment of contaminated water. Usually, the effluents are disinfected using oxidative processes, where chlorine is the most commonly used disinfectant [44]. Ozonation and UV irradiation approaches are gaining significant attention and have been increasingly applied [45,46]. UV LEDs have been directly used in wastewater treatment plants to inactivate bacteria. It has been found that a 280 nm wavelength LED was effective in eliminating *E. coli*, inducing a 4 log reduction [47]. In another study at 190 nm wavelength, pulsed UV irradiation was applied, generating an 11 log_10_ reduction rate [48].

Nevertheless, these wavelengths are also considered harmful to humans; thus, alternatives employing UV photoreactors have been explored. A 3D-printed UV reactor was developed, in which, similarly to our photoreactor, the lamps do not come in contact with the water, thus avoiding fouling processes and inducing a 6.9 log_10_ reduction in *E. coli* [49]. Despite the exciting results, this work aimed not to develop profound studies on optimising the disinfection process or its mechanism; several other studies focused on this topic [35,50,51]. Instead, the primary goal of the antimicrobial assays was to demonstrate the practicability and versatility of the developed photoreactor in disinfection studies. The benefit of the developed systems is very similar to the advantage enunciated in the CIP photocatalytic degradation tests, the possibility of adjusting radiation type and intensity, the agitation rate, reproducibility, and standardisation of the obtained results. This is particularly relevant for lab-scaled assays where the first assessments require standardisation to allow for conclusive results and straightforward comparisons between different antimicrobial efficiencies. 

## 4. Conclusions

Water remediation is a relevant area in the framework of the sustainable goals identified by the United Nations. Humankind’s future relies on our ability to develop new materials and devices to reestablish the water and natural environments to their original state. The possibility of having a multifunctional, portable, and efficient photoreactor will certainly leverage all the studies and outcomes in this area as it allows for the creation of experimental conditions that can be accurately controlled and reproduced in any laboratory or field using this device. 

In this work, we report on a new photoreactor based on concepts of portability, the possibility of using UV or visible radiation, incorporated agitation, and stability. The device was assembled by creating all the electronics, irradiation set-up, mechanical agitation arm, and an external FDM 3D-printed case. 

The portable device permits radiation intensities in an adjustable range between 7.5 and 110 mW/cm^2^ for UV radiation and 14 to 66 mW/cm^2^ for visible radiation, permitting studies regarding the radiation impact in photocatalytic and disinfection processes. Also, the agitation and refrigeration proved suitable and stable during lengthy irradiation assessments, which are paramount in the context of persistent contaminants.

Concerning the application assessments and the device proof of concept, the photocatalytic photoreactor proved to degrade 30% and 80% of ciprofloxacin under UV and visible radiation and eliminate *E. coli* efficiently. 

Compared to other photoreactors, this aesthetic device offers a simple, lightweight, and multifunctional approach with open-loop control, portability, and miniaturisation. It is convenient for swift laboratory photocatalytic assays and cost-effective actuation as a starting point for more standardised photocatalytic and disinfection experiments. The application of this photoreactor in a new lab or field combined with new photocatalysts and different water matrixes will be explored in the future based on the versatility of this device. 

## Figures and Tables

**Table 1:** Works applying TiO_2_-based materials to degrade different contaminants, the degradation reaction rate (*k*), and the type of radiation used.

Reactor Type	Photocatalytic Material	Contaminant	Degradation Efficiency (%)	Degradation Rate *k* (min^−1^)	Radiation Source	Reference
Slurry	TiO_2_	Ciprofloxacin	78	0.024	8 UV LEDs (365 nm)	Present work
Immobilised	TiO_2_	Ciprofloxacin	75	0.009	Irradiance 100 Wm^−2^ 365 nm	[40]
Slurry	TiO_2_	Metronidazole	98	0.022	UV lamp (9 W)	[17]
Slurry	TiO_2_	Roxithromycin	99	0.008	6 lamps (8 W each) at 350 nm	[41]
Slurry	Ag/TiO_2_	Ciprofloxacin	31	0.002	8 VIS LEDs (535 nm)	Present work
Immobilised	TiO_2_/AuNRs 650°	Nalidixic acid	65	0.001	QTH lamp (0.22 W/cm^2^~2 SUN	[42]
Immobilised	TiO_2_/GO	Ciprofloxacin	91	0.002	300 W xenon lamp with a 420 nm	[5]

## Data Availability

Data are contained within the article.
